# Temozolomide-Induced Shrinkage of a Pituitary Carcinoma Causing Cushing's Disease — Report of a Case and Literature Review

**DOI:** 10.1100/tsw.2010.210

**Published:** 2010-11-04

**Authors:** Lorenzo Curtò, Maria L. Torre, Francesco Ferraù, Vincenzo Pitini, Giuseppe Altavilla, Francesca Granata, Marcello Longo, Leo J. Hofland, Francesco Trimarchi, Salvatore Cannavò

**Affiliations:** ^1^ Department of Medicine and Pharmacology, Section of Endocrinology, University of Messina, Italy; ^2^ Department of Medical Oncology, University of Messina, Italy; ^3^ Department of Radiological Sciences, University of Messina, Italy; ^4^ Department of Internal Medicine, Division of Endocrinology, Erasmus Medical Center, Rotterdam, The Netherlands

**Keywords:** temozolomide, pituitary carcinoma, chemotherapy, O6-methylguanine DNA methyltransferase, Cushing’s disease

## Abstract

Temozolomide (TMZ) is an alkylating chemotherapeutic agent that has recently been used in some cases as a new therapeutic tool for pituitary carcinomas and aggressive pituitary adenomas. In this report, we present the case of effective TMZ treatment in a 42-year-old man with ACTH-secreting carcinoma. The tumor grew progressively over 4 years, from 2.2 to 31.1 cm^3^, despite three surgical approaches and gamma-knife treatment. Ki-67 increased from 2 to 18%. An intradural metastasis at the foramen magnum was detected by MRI after the third operation. Thereafter, four cycles of 5-day TMZ administration (200 mg/m^2^/day during the first, and 150 mg/m^2^/day during the following cycles) induced dramatic tumor size reduction (>90%). Clinical conditions improved progressively and, after 17 months from the beginning of TMZ administration, the patient is still alive. The treatment was well tolerated except for a transient thrombocytopenia (grade 4 WHO).

## INTRODUCTION

Pituitary carcinomas are extremely rare, representing no more than 0.1–0.2% of all pituitary tumors[1,2,3]. Most of them are diagnosed as prolactin (PRL)- or adrenocorticotropin (ACTH)-secreting invasive macroadenomas, but show aggressive growth characteristics and a propensity to metastasize with time[2,4,5,6]. Some reports suggested that silent ACTH-secreting macroadenomas, which cause signs or symptoms due to mass effect rather than Cushing's disease, are at increased risk of malignant transformation[7]. Corticotroph carcinomas, including those that are not active, account for 40% of all pituitary carcinomas[2,4,8]. The mechanisms underlying transformation of adenomas into carcinomas are not fully understood, and reliable predictive markers for transformation have not been identified to date[9,10]. Prognosis is poor in most cases, as surgery, radiotherapy, or drugs currently employed in the treatment of pituitary adenomas are not effective in controlling tumor growth and occurrence of metastases. Temozolomide (TMZ), an alkylating cytostatic drug introduced for treatment of glioblastoma multiforme, but effective in other CNS neoplasms as well as in neuroendocrine tumors, has also recently been proposed for management of pituitary carcinomas and aggressive pituitary adenomas[11,12,13,14]. This drug can alkylate and methylate specific guanine residues, damaging DNA and triggering the death of tumor cells. Effectiveness of TMZ is related to the down-expression of O-6-methylguanine-DNA methyltransferase (MGMT), an enzyme able to repair this type of DNA damage[12,15].

To date, only 15 cases of pituitary carcinomas treated with TMZ have been reported. We present the thirteenth case of effective TMZ treatment in patients with pituitary carcinoma, the fifth concerning a patient with ACTH-secreting malignant tumor. In our patient, TMZ induced a dramatic shrinkage of the tumor with stabilization of the intradural metastasis, after four cycles of treatment only, and dramatic improvement in clinical condition.

## CASE REPORT

A 42-year-old man was referred to our outpatient clinic in November 2004 with dramatic signs and symptoms of hypercortisolism, including diabetes mellitus, and widespread osteoporosis causing recurrent spontaneous rib fractures over 5 years. Physical examination showed abdominal obesity (BMI 32 kg/m$^2$), skin hyperpigmentation, edema of the lower limbs, livid striae on the abdomen, and severe hypertension. Endocrine investigation is summarized in Table 1. MRI showed an invasive pituitary mass extending to the suprasellar region with expansion into the left cavernous sinus (2.2 cm$^3$). After a trans-sphenoidal microscopic approach, which allows a subtotal resection of the tumor, histology revealed a sparsely granulated, PAS-negative, chromophobe adenoma, characterized by solid/diffuse architecture and rare mitotic figures. No Crooke’s hyaline change was evident. Immunohistochemistry was positive for ACTH in 70% of cells and tumor proliferation activity, calculated by the percentage of cells expressing Ki-67 antigen, was 2%. Immunohistochemical staining for the Ki-67 antigen was performed using the MIB-1 antibody and the avidin-biotin complex method using paraffin-embedded blocks cut into 3- to 4-μm-thick sections. CSF leak did not occur. After surgery, despite the persistence of a wide remnant, the patient experienced severe hypoadrenalism. Endocrine evaluation revealed undetectable plasma ACTH and low serum cortisol levels (5.5 μg/dl) (see Table 1). For this reason, hydrocortisone replacement was started. Six months later, stereotactic radiosurgery was performed by the gamma-knife (γ-knife) approach (21 Gy, in a single radiation dose) given tumor size increase (7.2 cm$^3$). This treatment induced mild tumor shrinkage, but after 2 years, MRI demonstrated tumor regrowth (27.0 cm$^3$), which spread into the left cavernous sinus and extended to the suprasellar and omolateral parasellar region with encasement of left internal carotid artery. Moreover, the patient complained of severe, subcontinuous headaches and dramatic decrease of visual acuity in the left eye. Thereafter, left cranial orbitozygomatic craniotomy with extensive resection of the pituitary tumor was performed. Pathology showed increased mitotic activity, bone invasion, and prominent cytological atypia. Immunohistochemistry was focally and weakly positive for ACTH. A nonspecific immunopositivity for growth hormone (GH) was also found. Moreover, Ki-67 index increased to 4%, while p53 was negative. However, soon after surgery, a huge tumor remnant was still evident and progressive tumor size increase was demonstrated by MRI after 1 year. For this reason, the patient underwent further subtotal resection of the tumor remnant by left craniotomy. Immunohistochemistry was focally positive only for ACTH and demonstrated that Ki-67 index was increased dramatically (18%). Six months after, in December 2008, the pituitary tumor continued to grow (31.1 cm$^3$) and an asymptomatic intradural lesion was detected by MRI (Fig. 1A–C). Histological examination of this mass was proposed, but the patient, informed about the risk of a surgical approach, did not consent to the biopsy. However, its imaging characteristics were consistent with a metastasis, as demonstrated by high-dose contrast MRI. Therefore, in February 2009, following the

**TABLE 1**
**Endocrine Evaluation at Presentation and after First Surgery**

| Basal and Dynamic Evaluation of the Hypothalamic-Pituitary-Adrenal Axis | At Presentation | After Surgery |
|---|---|---|
| Morning ACTH (pg/ml) | 93.0 (normal range 5.0–50.0) | Undetectable |
| Morning cortisol (µg/dl) | 44.3 (normal range 5.0–25.0) | 5.5 |
| Urinary free cortisol (µg/24 h) | 367.6 (normal range 5.0–72.0) | |
| ACTH after high-dose dexamethasone§ | 69.0 | |
| Cortisol after high-dose dexamethasone§ | 27.8 | |
| CRH (i.v. 1 µg/kg) stimulated ACTH (pg/ml) | 118 (peak = 143) | 14.6 (peak 17.9) |
| CRH (i.v. 1 µg/kg) stimulated cortisol (µg/ml) | 41 (peak = 45.2) | 5.5 (peak 6.7) |
| DDAVP* (i.v. 10 µg) stimulated ACTH (pg/ml) | 82 (peak = 378) | |
| DDAVP* stimulated cortisol (µg/ml) | 35.7 (peak = 63.4) | |

§ Oral dexamethasone suppression test (8 mg daily for 2 days, consecutively).

* Desmopressin acetate.

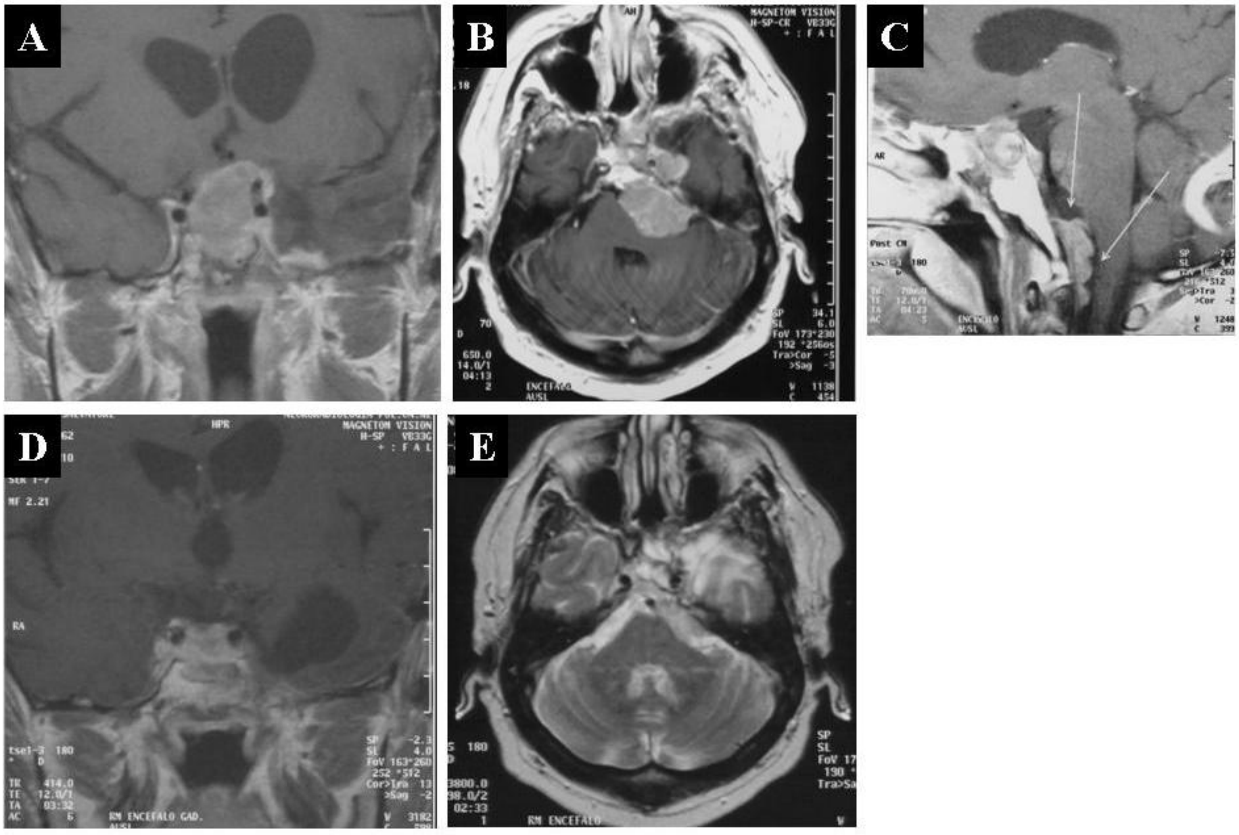

**FIGURE 1.** MRI evaluation before (A–C) and after (D,E) TMZ treatment. Before TMZ administration, tumor volume, investigated by sagittal, coronal (A), and axial (B) fast spin-echo (FSE) T1-weighted imaging after gadolinium-diethylenetriamine penta-acetate (Gd-DTPA) administration, was 31.1 $cm^3$. Sagittal (C) FSE T1-weighted imaging after Gd-DTPA administration demonstrated the presence of an intradural metastasis at the foramen magnum. After four TMZ cycles, tumor volume, investigated by sagittal and coronal (D) FSE T1-weighted imaging and by axial (E) FSE T2-weighted imaging after Gd-DTPA administration, was 2.8 $cm^3$.

demonstration of low MGMT expression (<5% nuclear staining) in specimens recovered from the second surgery, TMZ was administered orally at a daily dose of 200 mg/m$^2$ of body surface, for 5 days. This treatment was well tolerated except for a transient thrombocytopenia (grade 4 WHO) that required platelet transfusion. Seven weeks after the first cycle, TMZ was administered again at a lower daily dose (150 mg/m$^2$) for 5 days. Afterwards, MRI demonstrated dramatic tumor size shrinkage (5.2 cm$^3$), without changes of the intradural metastasis, and the patient experienced a decided lessening of headaches. Thus, a further two TMZ cycles, at a daily dose of 150 mg/m$^2$ for 5 days, were repeated every 4 weeks. The MRI performed 6 months after the end of TMZ treatment showed a further decrease of tumor size (2.8 cm$^3$) and a slight reduction of the intradural metastasis (Fig. 1D,E). Clinical conditions improved progressively and, after 17 months from the beginning of TMZ administration, the patient is still alive. At present, the hypoadrenalism did not recover, while the remaining pituitary function did not change compared with the evaluation performed before TMZ treatment (see Table 2).

**TABLE 2**
**Endocrine Evaluation Before and After TMZ Treatment**

| | Before TMZ | After TMZ |
|---|---|---|
| Follicle-stimulating hormone (FSH) | 3.87 U/L (normal range 0.7–11.1) | 3.7 |
| Luteinizing hormone (LH) | 4.67 U/L (normal range 0.8–7.6) | 3.8 |
| Total testosterone | 512 ng/dl (normal range 280–800) | 467 |
| Prolactin (PRL) | 260.6 U/L (normal range 86–324) | 200.4 |
| Thyroid-stimulating hormone (TSH) | 1.8 mU/L (normal range 0.27–4.2) | 2.0 |
| Free thyroxine (FT4) | 15.5 pmol/l (normal range 12–22) | 14.9 |
| Growth hormone (GH) | 0.7 ng/ml (normal range 0.2–2.0) | 0.8 |
| Insulin-like growth factor 1 (IGF-1) | 201 ng/ml (normal range 101–267) | 195 |
| Urinary free cortisol (UFC) | 37.5 μg/24 h (normal range 5.0–72.0) | 69.1[§] |

[§] Dosage of hydrocortisone replacement therapy was decreased after the last cycle of TMZ.

## DISCUSSION

Pituitary carcinomas are extremely rare[1,2,3] and are characterized by invasion of adjacent structures, rapid proliferation, and/or presence of distant metastases to intra- or extracranial sites[4,9]. In many cases, they are the result of the progressive transformation of an apparently benign, invasive macroadenoma. During malignant transformation, changes in hormone secretion have been reported in some cases, probably because these tumors were polyclonal, and able to express different morphology and biological activity during growth[4,7,16]. The demonstration of a different genetic pattern in the specimens collected when the patient in question was reoperated several times might support this hypothesis[14,17]. ACTH-secreting macroadenomas with low sensitivity to corticotropin-releasing hormone (CRH) or high-dose dexamethasone, or with low hormone secretion rate, so-called silent corticotropinomas, are at increased risk for invasiveness or transformation into carcinomas[18]. Conventionally, an invasive pituitary macroadenoma is considered carcinoma only when distant metastases occur[4]. More frequently, they are intracranial or spinal, and cause neurological symptoms[4]. Intra-abdominal or lung metastases have also been reported[2,4,19]. Owing to the low incidence of pituitary carcinomas, there is poor experience regarding treatment. Surgery, radiotherapy, and medical therapy with dopamine agonists, somatostatin analogs, tamoxifen, cyproheptadine, OP’DDD (mitotane), or systemic chemotherapy have been proposed in many cases, but effects are inconstant or palliative[4,10,20,21,22,23,24] and a prolongation of patients’

long-term survival was not reported[20,25]. On the contrary, the surgical excision of metastases seemed to improve the chances of survival in some cases[26]. Recently, TMZ has been introduced in the management of malignant primary neoplasms and metastases of the CNS[27,28,29]. This drug, specific for the treatment of brain tumors due to its ability to cross the blood brain barrier and a good liposolubility in brain tissue, has also been proposed for pituitary carcinomas and aggressive pituitary adenomas resistant to conventional management[11,12]. Until now, TMZ administration has been able to reduce tumor size and improve clinical conditions in a patient with LH-secreting pituitary carcinoma, in four patients with PRL-secreting carcinoma, in a patient with mixed GH- and PRL-secreting carcinoma, and in three patients with ACTH-secreting pituitary carcinoma[11,12,15,30,31,32,33,34,35]. Fadul et al.[11] also reported a patient with PRL-secreting carcinoma in whom TMZ induced improvement of clinical condition, dramatic PRL level decrease, and tumor volume reduction at the beginning of treatment. Nevertheless, discontinuation of TMZ was followed, 15 months after, by increased PRL levels and metastasis recurrence. More recently, a significant tumor shrinkage with a decreased hormone secretion was reported by Raverot et al. in two out of five patients with pituitary carcinoma (one with ACTH- and another with PRL-secreting carcinoma) included in a French multicenter study[13]. In these patients, the efficacy of TMZ was demonstrated after only three cycles of treatment. Other studies reported several cases with invasive secreting or nonfunctioning pituitary adenomas effectively treated with this drug[12,13,36,37,38,39,40]. TMZ promotes apoptosis of target cells, causing alkylation and methylation of guanine residues in DNA, and induces massive cell shrinkage and necrosis. Its effectiveness is related to the tumor down-expression of MGMT, an enzyme able to repair this type of DNA damage[12,15]. Accordingly, high MGMT expression was reported in a nonfunctioning pituitary adenoma not responding to TMZ[41] and in a case of aggressive ACTH-secreting pituitary adenoma that transformed into a carcinoma 4 months after TMZ withdrawal[34]. On the contrary, MGMT expression was high in an ACTH-secreting carcinoma responding, and low in a PRL-secreting carcinoma and in an aggressive ACTH-secreting adenoma not responding, to TMZ[13]. Moreover, a marked heterogeneity in MGMT expression was found in many cases, with a predominant expression both in single regions and in the periphery of the tumor[40]. For this reason, MGMT status should be considered a poor predictor of treatment outcome and it should not be used for the selection of patients candidate to TMZ therapy.

In our patient, the treatment induced a dramatic lessening of headaches. After four TMZ cycles, pituitary tumor volume was reduced by more than 90% and a stabilization of the metastasis volume was documented. The drug was well tolerated and transient thrombocytopenia, managed by platelet transfusion, was the only side effect that occurred after the first cycle of treatment.

In conclusion, TMZ can be considered an effective and safe approach to aggressive pituitary tumors. Moreover, a short trial with TMZ could be useful to identify potential responder patients in comparison with the MGMT expression.
